# Supplementary material for: Vitamin D Modified DSS-Induced Colitis in Mice via STING Signaling Pathway
Source: Biology (Basel). 2025 Jun 18;14(6):715. doi: 10.3390/biology14060715 (PMC12190092; doi:10.3390/biology14060715)
Supplement: Supplementary file 1 [file biology-14-00715-s001.zip › Supplementary Materials S1: 16s-report of gut microbiota/Groups/diff_analysis/RF/OTU_Groups.RFimportance.pdf]

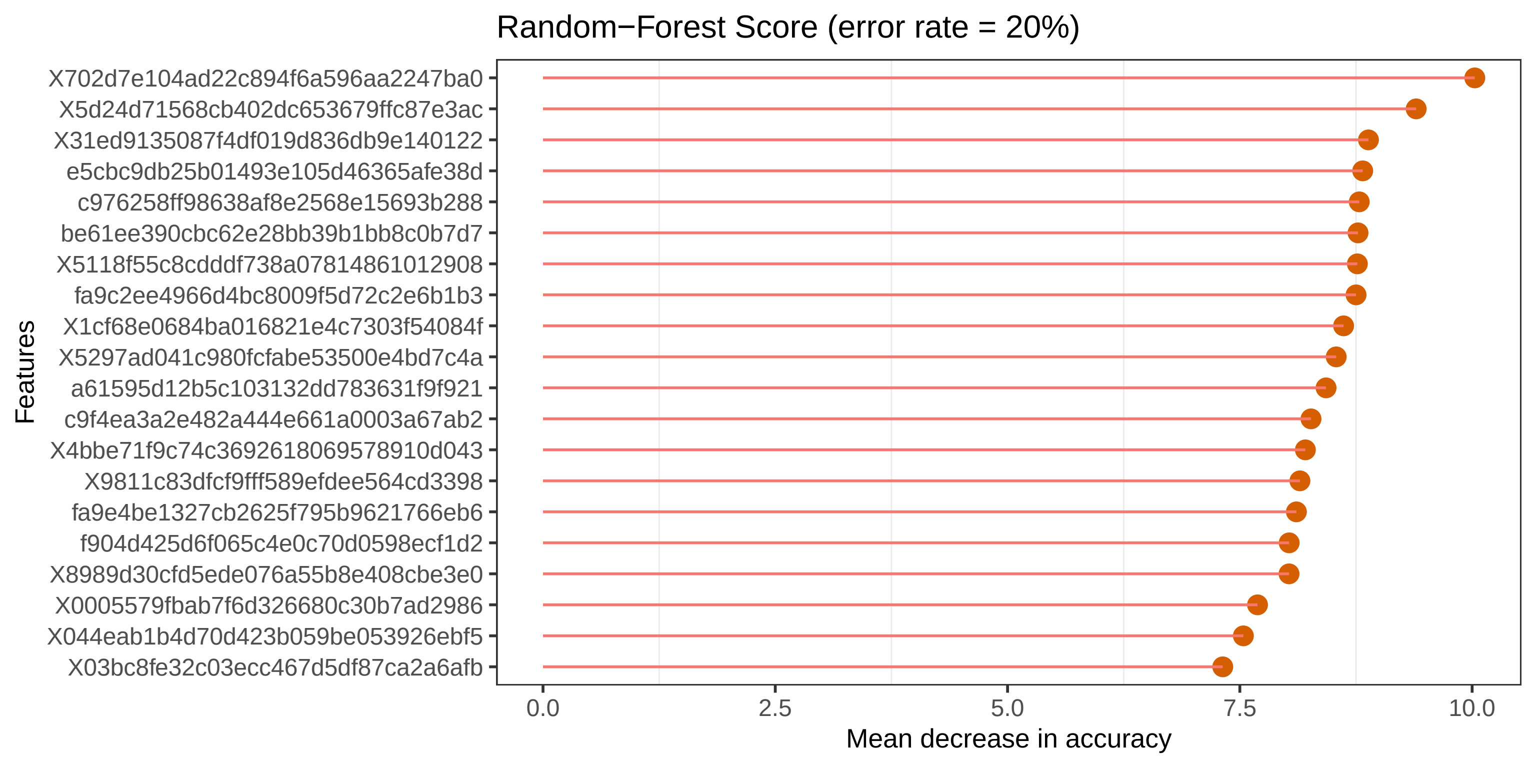
Random−Forest Score (error rate = 20%)
Features
X702d7e104ad22c894f6a596aa2247ba0
X5d24d71568cb402dc653679ffc87e3ac
X31ed9135087f4df019d836db9e140122
e5cbc9db25b01493e105d46365afe38d
c976258ff98638af8e2568e15693b288
be61ee390cbc62e28bb39b1bb8c0b7d7
X5118f55c8cdddf738a07814861012908
fa9c2ee4966d4bc8009f5d72c2e6b1b3
X1cf68e0684ba016821e4c7303f54084f
X5297ad041c980fcfabe53500e4bd7c4a
a61595d12b5c103132dd783631f9f921
c9f4ea3a2e482a444e661a0003a67ab2
X4bbe71f9c74c36926180695789 10d043
X9811c83dfcf9fff589efdee564cd3398
fa9e4be1327cb2625f795b9621766eb6
f904d425d6f065c4e0c70d0598ecf1d2
X8989d30cfd5ede076a55b8e408cbe3e0
X0005579fbab7f6d326680c30b7ad2986
X044eab1b4d70d423b059be053926ebf5
X03bc8fe32c03ecc467d5df87ca2a6afb
0.0
2.5
5.0
7.5
10.0
Mean decrease in accuracy
